# Using mass spectrometry to validate mouse models of tauopathy

**DOI:** 10.1186/s13024-023-00615-6

**Published:** 2023-04-05

**Authors:** Yan Yan, Casey N. Cook

**Affiliations:** 1 Department of Neuroscience, Mayo Clinic, Jacksonville, FL USA; 2 Neurobiology of Disease Graduate Program, Mayo Graduate School, Mayo Clinic College of Medicine, Rochester, MN USA

**Keywords:** Tau, Alzheimer’s disease, Frontotemporal dementia, Phosphorylation, Mouse model, Tauopathy, Mass spectrometry

Aggregation of the microtubule-binding protein tau is a neuropathological hallmark of a group of neurodegenerative disorders classified as tauopathies, including Alzheimer’s disease (AD) and frontotemporal dementia (FTD) [1, 2]. While pathogenic mutations in the tau gene (*MAPT*) have been linked to FTD and other primary tauopathies, no pathogenic *MAPT* mutations have been associated with AD. Despite this, mouse models of tauopathy typically rely on pathogenic mutations to promote tau accumulation, and are commonly described as models of AD given their ability to recapitulate Alzheimer’s-like, neurofibrillary tau pathology (reviewed [3]). Although there has been considerable debate regarding their suitability as models of AD, a thorough investigation of the extent to which common mouse models of tauopathy accurately recapitulate key features of AD was lacking. However, the recent generation of a comprehensive map of tau post-translational modifications (PTMs) in postmortem brain tissue from patients with AD compared to control individuals [4] provided the necessary framework to finally address this question. As such, published on February 2, 2023 in *Molecular Neurodegeneration*, Wenger and colleagues used mass spectrometry to characterize the tau PTM landscape in two widely utilized mouse models of tauopathy (hTau.P301S and rTg4510), and subsequently compared this profile with both FTD patients carrying P301L mutations [5] and AD patients using their previous dataset [4].

Prior to performing mass spectrometry, the authors first compared the levels of soluble and insoluble tau across aging in both hTau.P301S and rTg4510 models, which revealed that accumulation of insoluble tau precedes tangle formation in both models. Moreover, the abundance of insoluble tau in aged rTg4510 mice (but not hTau.P301S mice) is comparable to the amount of tau accumulation in human AD patients (Braak stage IV-V). Given the different promoters used to drive transgene expression in the hTau.P301S and rTg4510 models, as expected the authors also observe differences in affected brain regions. Specifically, the brainstem shows the earliest increase in insoluble tau followed by the cortex and subcortical regions in the hTau.P301S model, which uses a Thy-1 promoter to drive human mutant tau expression. In contrast, tau accumulation was first observed in the cortex and hippocampus in rTg4510 mice, which is a bigenic model that relies on the presence of a P301L tau transgene and a tetracycline transactivator (tTA) transgene driven by the CaMKIIa promoter to conditionally express mutant human tau in the forebrain [6].

Following the biochemical characterization of tau deposition, Wenger and associates utilized a quantitative mass spectrometry method called FLEXITau (full-length expressed stable isotope-labeled Tau)[7] to generate a tau PTM map across aging and brain region in both mouse models. While tau phosphorylation, citrullination, methylation and ubiquitination were observed in both models, phosphorylation was identified as the main PTM driving tau aggregation. Specifically, phosphorylation was identified in the cortex as early as 1.5-2 months of age in both mouse models, with a progressive accumulation that positively correlated with insoluble tau levels in the brain. In addition, some phosphorylation sites were only observed in the presence of pathology, including pT212, pS214, pT217 and pT403. These results suggest that hypermodified, hyperphosphorylated tau is the primary PTM driving tauopathy progression in the hTau.P301S and rTg4510 models.

Finally, to provide animal model validation and assess the extent to which hTau.P301S and/or rTg4510 mice accurately recapitulate AD versus familial FTD, as well as to elucidate differences in the tau PTM profile between these different forms of tauopathy, Wenger and colleagues examined tau PTMs in human FTD patients carrying the *MAPT* P301L mutation (FTD/P301L). Similar to the tau PTM landscape in hTau.P301S and rTg4510 mice, phosphorylation was the predominant modification of insoluble tau in FTD/P301L patients. In addition, although the majority of tau peptides were modified in FTD/P301L patients compared to controls, the modification extent was overshadowed by the level achieved in both the hTau.P301S and rTg4510 models. Of particular relevance, regions within the proline-rich domain and C-terminus of tau represented the most highly-modified in FTD/P301L and AD patients, as well as both mouse models. Moreover, although tau ubiquitination and acetylation on lysine residues is characteristic of late-stage AD, these modifications were largely absent from both FTD/P301L patients and mouse models (with the exception of tau ubiquitination in the first microtubule-binding repeat domain detected in rTg4510 mice with aging). Based on these results, the authors conclude that the hTau.P301S and rTg4510 models recapitulate disease in FTD/P301L and early-stage AD, in which tau aggregation is driven primarily by phosphorylation.

Overall, there are several important implications of this work. Most tangible is the comprehensive tau PTM map constructed by the authors that will guide future studies that incorporate the hTau.P301S and rTg4510 mouse models of tauopathy. This study also provides a framework that can be extended to include and validate additional models of tauopathy. One of the overall goals of the study was to assess whether drug candidates that demonstrated preclinical efficacy in either the hTau.P301S or rTg4510 may have failed in clinical trials due to an inability of the models to accurately recapitulate human disease. Therefore, the discovery that the tau PTM landscape in hTau.P301S and rTg4510 mice is similar to that observed in FTD/P301L patients and early-stage AD may suggest: (1) targets demonstrating preclinical efficacy in hTau.P301S or rTg4510 mice may demonstrate therapeutic benefit in FTD/P301L patients (despite potential failures in clinical trials in patients with late-stage AD); (2) therapeutic targets identified in hTau.P301S and rTg4510 mice may be suitable for early-stage AD (dominated by phosphorylation) but not late-stage AD (increase in lysine PTMs that are predominantly absent in mouse models). However, potentially most intriguing is the comparison of tau PTM profiles between FTD/P301L and AD patients, with the most similarities observed with early-stage but not late-stage AD. As such, future studies will be needed to identify the specific pathways and mechanisms that drive extensive modification of tau’s lysine residues in late-stage AD, which appear to be absent in FTD/P301L patients.

## Data Availability

Not applicable.

## Abbreviations

AD: Alzheimer’s disease
FLEXITau: full-length expressed stable isotope-labeled tau
FTD: frontotemporal dementia
MAPT: microtubule-associated protein tau
PTM: post-translational modification
